# The rRNA epitranscriptome and myonuclear SNORD landscape in skeletal muscle fibers contributes to ribosome heterogeneity and is altered by a hypertrophic stimulus

**DOI:** 10.1152/ajpcell.00301.2024

**Published:** 2024-06-24

**Authors:** Minying Cui, Paulo Jannig, Maral Halladjian, Vandré C. Figueiredo, Yuan Wen, Ivan J. Vechetti, Nicolai Krogh, Baptiste Jude, Sebastian Edman, Johanna Lanner, John McCarthy, Kevin A. Murach, Thomas Sejersen, Henrik Nielsen, Ferdinand von Walden

**Affiliations:** ^1^Department of Women’s and Children’s Health, Karolinska Institutet, Stockholm, Sweden; ^2^Department of Cellular and Molecular Medicine, University of Copenhagen, Copenhagen, Denmark; ^3^Department of Biological Sciences, Oakland University, Rochester, Michigan, United States; ^4^Department of Physiology, College of Medicine, University of Kentucky, Lexington, Kentucky, United States; ^5^Center for Muscle Biology, University of Kentucky, Lexington, Kentucky, United States; ^6^Division of Biomedical Informatics, Department of Internal Medicine, College of Medicine, University of Kentucky, Lexington, Kentucky, United States; ^7^Department of Nutrition and Health Sciences, University of Nebraska-Lincoln, Lincoln, Nebraska, United States; ^8^Department of Physiology and Pharmacology, Karolinska Institutet, Stockholm, Sweden; ^9^Department of Health, Human Performance, and Recreation, Exercise Science Research Center, University of Arkansas, Fayetteville, Arkansas, United States; ^10^Department of Child Neurology, Karolinska University Hospital, Astrid Lindgren Children’s Hospital, Stockholm, Sweden; ^11^Center for Neuromusculoskeletal Restorative Medicine, Hong Kong Science Park, Hong Kong, People’s Republic of China

**Keywords:** epitranscriptomics, hypertrophy, 2′-O-Me, ribosome heterogeneity, skeletal muscle

## Abstract

In cell biology, ribosomal RNA (rRNA) 2′*O*-methyl (2′-*O*-Me) is the most prevalent posttranscriptional chemical modification contributing to ribosome heterogeneity. The modification involves a family of small nucleolar RNAs (snoRNAs) and is specified by box C/D snoRNAs (SNORDs). Given the importance of ribosome biogenesis for skeletal muscle growth, we asked if rRNA 2′-*O*-Me in nascent ribosomes synthesized in response to a growth stimulus is an unrecognized mode of ribosome heterogeneity in muscle. To determine the pattern and dynamics of 2′-*O*-Me rRNA, we used a sequencing-based profiling method called RiboMeth-seq (RMS). We applied this method to tissue-derived rRNA of skeletal muscle and rRNA specifically from the muscle fiber using an inducible myofiber-specific RiboTag mouse in sedentary and mechanically overloaded conditions. These analyses were complemented by myonuclear-specific small RNA sequencing to profile SNORDs and link the rRNA epitranscriptome to known regulatory elements generated within the muscle fiber. We demonstrate for the first time that mechanical overload of skeletal muscle *1*) induces decreased 2′-*O*-Me at a subset of skeletal muscle rRNA and *2*) alters the SNORD profile in isolated myonuclei. These findings point to a transient diversification of the ribosome pool via 2′-*O*-Me during growth and adaptation in skeletal muscle. These findings suggest changes in ribosome heterogeneity at the 2′-*O*-Me level during muscle hypertrophy and lay the foundation for studies investigating the functional implications of these newly identified “growth-induced” ribosomes.

**NEW & NOTEWORTHY** Ribosomal RNAs (rRNAs) are posttranscriptionally modified by 2′*O*-methyl (2′-*O*-Me). This study applied RiboMeth-seq (RMS) to detect changes in 2′-*O*-Me levels during skeletal muscle hypertrophy, uncovering transient diversification of the ribosome pool in skeletal muscle fibers. This work implies a role for ribosome heterogeneity in skeletal muscle growth and adaptation.

## Graphical abstract

**Figure:**
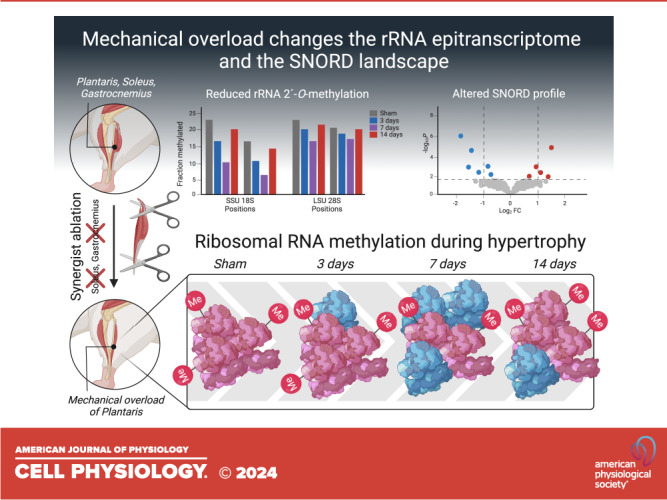

## INTRODUCTION

Ribosome biogenesis is essential for cellular homeostasis and is a complex process with numerous regulated steps. Skeletal muscle RNA is 80–85% ribosomal RNA (rRNA), and skeletal muscle growth via mechanical overload (MOV) increases ribosome biogenesis rates in humans and rodents (1). Recent data in human skeletal muscle suggest that the proportion of nascent ribosomes rather than total amount of ribosomes quantified early during the hypertrophic process influences accretion of mass several weeks later (2). These new ribosomes may differ in composition and function relative to pre-existing ribosomes (2). Studies in nonmuscle cell types reveal that ribosome composition is far more heterogeneous than first anticipated (3–7). This heterogeneity influences translation control by, for example, modulating translation fidelity, translation speed, and/or by influencing mRNA selectivity (3, 4). In skeletal muscle, ribosomes may be specialized by MOV at the ribosomal protein level. Following MOV, mRNA abundance of the striated muscle-specific ribosomal protein-paralogue Rpl3-like (RPL3L) drops dramatically and is replaced by the more ubiquitously expressed RPL3 (8). Moreover, overexpression of RPL3L during differentiation of myotubes in vitro results in thinner myotubes with a reduced protein content (8). These experiments suggest that ribosome specialization potentially represents an underappreciated but necessary role during skeletal muscle hypertrophy (8).

During ribosome biogenesis, recent evidence indicates that rRNAs are heavily modified posttranscriptionally with pseudouridines, methyls, and other chemical modifications (9). Ribose 2′-*O*-methyl (2′-*O*-Me) is the most prevalent type of rRNA modification (∼112 modifications on human and mouse rRNA) (10, 11). The methyltransferase fibrillarin (Fbl) installs 2′-*O*-Me and modification is mediated by a family of small nucleolar RNAs (snoRNAs) (12). Fibrillarin is guided to specific rRNA sites by box C/D snoRNAs (SNORDs) (13) that, in a precise manner, targets the sites to be methylated in pre-rRNA by forming a transient duplex structure (14). Variations of these modification result in specialized ribosomes promoting preferential translation of specific mRNAs (7, 15), sufficient to drive cell fate decisions (16, 17). However, an individual site along the rRNA can only be methylated or not methylated in the mature ribosome and the addition of a methyl group on rRNA is considered irreversible. The current study addressed whether the skeletal muscle rRNA methylation pattern is influenced by MOV, indicative of contribution by nascent ribosomes with a specialized 2′-*O*-Me pattern.

We used RiboMeth-seq (RMS) to determine the influence of MOV on the 2′-*O*-Me pattern of skeletal muscle rRNA. In addition to performing experiments using an inducible muscle-specific RiboTag mouse, we performed transcriptomic analysis of myonuclear-specific SNORD expression and linked these data to the observed 2′-*O*-Me change. The results of our study provide the first description of 2′-*O*-Me in skeletal muscle fibers and how rRNA methylation changes at the level of the ribosome pool in response to a growth stimulus. We provide new research avenues for exploring ribosome heterogeneity in skeletal muscle and how rRNA methylation may contribute to the adaptive response.

## MATERIALS AND METHODS

### Animal Experiments

All experiments were approved by the Institutional Animal Care and Use Committee (IACUC) of the University of Kentucky. Mice were housed in a temperature- and humidity-controlled room, maintained on a 14:10-h light-dark cycle, and food and water were provided ad libitum throughout experimentation. Animals were humanely euthanized via CO_2_ asphyxiation followed by cervical dislocation.

Samples from two previously published mouse experiments (*experiments 1* and *2*) were used in the current study (18, 19).

#### Experiment 1.

Whole skeletal muscle RNA following 3, 7, and 14 days of MOV and a sham surgery control condition in C57BL/6J (females, 5 mo of age, *n* = 12) were used for RiboMeth-seq analysis (described in the *RiboMeth-Seq Analysis* section). MOV was induced by synergist ablation for which a small portion of the gastrocnemius muscle was removed (20), whereas sham surgery involved the same steps of synergist ablation without the removal of muscle.

#### Experiment 2.

Myonuclear RNA was isolated from 3-day overload/sham female HSA-GFP mice (HSA-rtTA+/−;TRE-H2B-GFP+/−, experimental details previously published) (21, 22). In brief, mice (females, ∼3 mo of age, sham *n* = 3 and MOV *n* = 4) were given doxycycline (0.5 mg/mL, 2% sucrose) for 5 days followed by a minimum 1-wk washout period prior to MOV (3 wk maximum). This strategy leads to the labeling of ∼90–95% of myonuclei, with negligible off-target labeling of nonmyonuclei (22).

#### Experiment 3.

A skeletal muscle-specific RiboTag mouse (HSA-RiboTag) was generated by crossing the HSA-mER-CRE-mER (MCM) mouse (Jax, Stock No. 025750) with the RiboTag mouse (Jax, Stock No. 029977), which enabled cre-mediated hemagglutinin (HA) epitope tagging of ribosomes at the ribosomal protein L22 locus in a skeletal muscle-specific manner. HSA-RiboTag mice (females, 4–6 mo of age, *n* = 3 overload/sham) were administered tamoxifen (75 mg/kg body wt, corn oil solution) by intraperitoneal injection for 5 days starting on the day of MOV. For this experiment, MOV was performed on one limb with the contralateral limb serving as sham control.

### RiboTag Immunoprecipitation

Plantaris muscles were homogenized with a handheld Dounce homogenizer (RNAse-free) in polysome buffer [20 mM Tris pH 7.4, 10 mM MgCl, 250 mM KCl, 2 mM DTT, 1% Triton X-100, 100 µg/mL cycloheximide (CHX)]. The lysates were rotated at 4°C for 10 min before centrifugation at 10,000 *g* 10 min. The supernatants were retained for immunoprecipitation (IP). To perform IP, anti-HA antibody (H6908, Sigma) was added to the lysate and incubated at 4°C on a rotator for 6 h. Then, 50 µL of magnetic beads (Dynabeads Protein G, Thermo Fisher Scientific) were added to the lysate-antibody mix and incubated overnight. Prior to incubation, beads were washed three times in polysome buffer. After incubation, the lysate was removed and kept for RNA isolation. The beads were then washed three times with polysome buffer before the addition of TRI Reagent for RNA isolation.

### RNA Isolation

Whole tissue RNA from plantaris muscles was extracted using TRI Reagent (Sigma-Aldrich, St. Louis, MO). Skeletal muscle tissue samples were homogenized using beads and the Bullet Blender tissue homogenizer (Next Advance, Troy, NY). Thereafter, RNA was isolated via phase separation (bromochloropropane and centrifugation). RNA was extracted from the supernatant using the Direct-zol kit (Zymo Research, Irvine, CA) with on-column DNase treatment, eluted in nuclease-free water.

HSA-GFP mouse plantaris muscles were Dounce homogenized and subjected to fluorescence-activated nuclei sorting (FANS) for purification of myonuclei according to our published protocols (21, 23–25). RNA from myonuclei was isolated as previously reported (21, 23).

### RiboMeth-Seq Analysis

RiboMeth-seq (RMS) was performed as previously reported (26) with only minor modifications. In brief, 5 μg of whole cell RNA was fragmented under denaturing conditions (90°C) by incubation in alkaline buffer (pH 9.9) for 5 min. Then, the RNA was size fractionated on a denaturing (urea) polyacrylamide gel, and fragments in the size range 20–40 nt were excised and eluted. The fragments were ligated to adapters using a modified tRNA ligase, and cDNA were synthesized using Superscript IV (Thermo Fisher Scientific) to form a fragment library for sequencing using the Ion Proton platform. Reads were mapped to a curated mouse rDNA reference sequence and SNORDs. RMS scores (fraction methylated) were calculated as “score C” as in the study by Birkedal et al. (27). In a few cases, a barcode correction was applied when calculating the RMS score as described previously (28). The number of sequencing reads mapping to specific RNAs was used as a measure of their levels and is presented as reads per kilobase of transcript, per million mapped reads (RPKM) ± SE. Statistical significance was determined using two-tailed Student’s unpaired *t* test (*P* < 0.05) in MOV conditions and paired *t* test (*P* < 0.05) in the HSA-GFP mouse (sham vs. MOV). For all analyses and conditions, a biological triplicate was used.

Heatmap was used to visualize the rRNA methylation changing pattern at different timepoints of MOV. The heatmap represented the hierarchical clustering of *Z*-score-normalized rRNA methylation scores. Hierarchical clustering identified clusters based on significant changes in rRNA sites between MOV conditions and sham (unpaired *t* test, *P* value < 0.05). Heatmap was produced in R using the pheatmap package (http://cran.r-project.org/web/packages/pheatmap/).

### Quantification of SNORDs by Small RNA Sequencing

Small RNA sequencing (RNA-Seq) was performed from the myonuclear RNA isolated from HSA-GFP mice plantaris (MOV *n* = 4 and sham surgery *n* = 3). A detailed workflow can be found in the previous report (18). Acquisition of raw counts through feature counts and use as input for the statistical analysis by DESeq2 (29) to identify differentially expressed genes (DEGs). All reads with less than 10 counts were removed from the analysis. DEGs identified with a false discover rate (Benjamini–Hochberg method) adjusted *P* value <0.05 and log2 fold change over 1. Enhanced volcano plot was used for sample visualizations and generated in R using the Bioconductor package. (https://bioconductor.org/packages/devel/bioc/vignettes/EnhancedVolcano/inst/doc/EnhancedVolcano.html).

### Codon Composition Analysis

Raw sequencing data from our previously published bulk RNA sequencing of mouse plantaris muscle after 72 h of mechanical overload (GSE213406) (21) were reanalyzed for transcript-level differential expression. Quality control preprocessing was performed with FastQC v. 0.11.9, followed by adapter and low-quality base removal using Cutadapt 4.4 and Trim Galore 0.6.10, respectively. Transcript quantification was performed by aligning preprocessed reads to the mouse transcriptome (GRCm38.06, release-102) using Kallisto (30) (v.0.46.1). DESeq2 (31) was used for differential expression analysis with adaptive log fold change shrinkage estimator from the ashr package (32), and the list of protein-coding transcripts was retrieved. Reactome pathways were obtained from the Molecular Signatures Database (MSigDB) (33) using the clusterProfiler R package (34), and overrepresentation analysis was performed using enricher function. Adjusted *P* values for enrichment tests were calculated with Benjamini–Hochberg procedure. Coding sequences were retrieved from Ensembl (GRCm38.p6, release-102) using biomaRt R package and getSequence function. Codon frequency and guanine-cytosine (GC) content calculations were performed excluding the start codon using seqinr R package. GC content for each transcript and median frequency of each codon per set of transcripts were calculated and compared with background transcripts (all detected transcripts). *P* values were calculated with Wilcoxon test and corrected for multiple comparison using Bonferroni. Code used is available in the GitHub repository paulojannig/RMS_muscle_overload.

## RESULTS

### Ribosomes with Hypomethylated rRNA at a Subset of Sites Are Transiently Present during Mechanical Overload-Induced Hypertrophy

**Figure 1.**
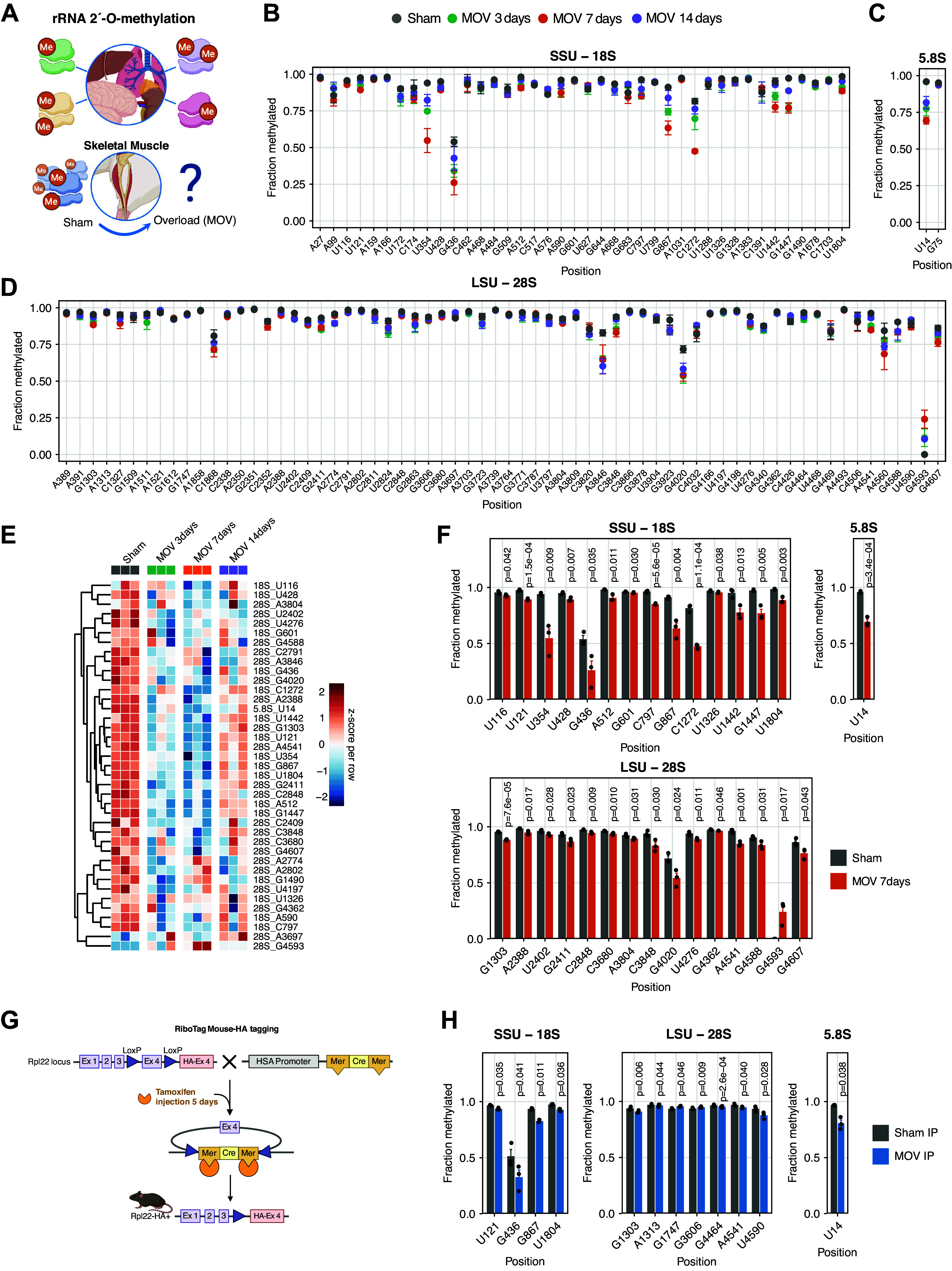
*A*: ribosomal RNA (rRNA) 2′-*O*-methyl (2′-*O*-Me) in skeletal muscle with mechanical overload (MOV). *B*–*D*: graph depicting methylation scores at all methylated positions in mouse rRNA at sham and at 3 different timepoints post MOV (3, 7, and 14 days, *n* = 3 each time point). About 108 known 2'-*O*-Me sites in mouse were analyzed cross all timepoints in biological triplicates. Sites (*x*-axis) are numbered according to human rRNA to allow for comparisons. The corresponding nucleotide positions for human and mouse rRNA can be found in Supplemental Table S1. Error bars indicate the standard deviation. LSU, large ribosomal subunit; SSU, small ribosomal subunit. *E*: heatmap depicting the hierarchical clustering of *Z*-score-normalized rRNA methylation scores. Clusters were identified by hierarchical clustering of significantly changed rRNA sites compared between different MOV timepoints and sham (two-tailed Student’s unpaired *t* test, *P* value <0.05). Rows represent significantly changed rRNA sites included in the clusters, and columns represent different timepoints of MOV (the biological replicates). Rows were clustered using Euclidean distance. *F*: graph showing rRNA sites with statistically significantly changed methylation scores with overload 7 days as compared with sham (*n* = 3, respectively). Two-tailed Student’s unpaired *t* test was used (*P* < 0.05). *G*: schematic figure of the HSA-RiboTag mouse and experimental setup. *H*: graph depicting the significant methylation sites from HSA-RiboTag mouse with HA-tagged ribosomes. Paired *t* test was used to compare the methylation scores in overload immunoprecipitation (IP) leg and sham IP leg (*n* = 3) (*P* value <0.05). Created with BioRender.

Considering the previously observed heterogeneity of rRNA 2′-*O*-Me patterns among and within various tissues and cell types (11, 17), we sought to determine whether a potent growth stimulus, such as the MOV model, could influence the skeletal muscle rRNA 2′-*O*-Me profile (Fig. 1*A*). MOV (synergist ablation) was performed for 3, 7, and 14 days in mice, and the plantaris muscle 2′-*O*-Me profile was determined using RiboMeth-seq and was compared with sham group (Fig. 1, *B–D*). Among a subset of significantly differentially methylated sites, there was a decreased methylation pattern between 3 and 7 days, which reverted to the sham methylation level by 14 days (Fig. 1*E*). The most pronounced decreased methylation was observed with overload at 7 days (Fig. 1, *B–E*), with 30 of 108 sites showing a statistically significant change in methylation (Fig. 1*F*). Specifically, we observed three different types of methylation changes: *1*) a sharp decreased methylation (≥0.15 difference in RMS score) [small ribosomal subunit (SSU)-U354, SSU-G436, SSU-G867, SSU-C1272, SSU-G1447, large ribosomal subunit (LSU)-G4020 and 5.8S-U14], *2*) rRNA sites with slightly decreased methylation (≤0.15 difference in RMS score) (SSU-U116, SSU-U121, SSU-U428, SSU-A512, SSU-G601, SSU-C797, SSU-U1326, SSU-U1442, SSU-U1804, LSU-G1303, LSU-A2388, LSU-2402, LSU-G2411, LSU-C2848, LSU-C3680, LSU-A3804, LSU-C3848, LSU-U4276, LSU-G4362, LSU-A4541, LSU-G4588, and LSU-G4607), and *3*) one rRNA site with markedly increased methylation from nonmethylation in sham (LSU-G4593).

### Myofiber-Specific Ribosomes Respond to Mechanical Loading by Changing 2′-O-Me Pattern

Next, we asked whether the observed rRNA 2′-*O*-Me changes following MOV is specific to myofibers or whether proliferating interstitial or infiltrating cells influence the observed response. To answer this, we used our myofiber-specific, inducible HSA-RiboTag mouse model and performed 7 days of unilateral MOV to enable isolation of myofiber-specific ribosomes (Fig. 1*G*). We first compared the whole muscle rRNA 2′-*O*-Me profile (sham group from Fig. 1, *B–D*) with myofiber-specific rRNA from HA-tagged ribosomes in the sham limb (sham IP) and observed that the 2′-*O*-Me profile in skeletal muscle, with few exceptions, reflects myofiber ribosomes (Supplemental Fig. S1, *A*–*C*; all Supplemental files are available at https://doi.org/10.6084/m9.figshare.25943965). Subsequently, when comparing sham IP and overload IP from the HSA-RiboTag mice, 12 differentially methylated sites (Fig. 1*H*) were observed (Supplemental Fig. S1*D*). We then compared the significantly changed sites from whole tissue RNA (MOV plantaris muscle, Fig. 1*F*) and isolated muscle fiber-specific ribosomes following MOV (HSA-RiboTag, Fig. 1*H*) to find overlapping sites for further downstream analysis. We identified seven overlapping sites (SSU-U121, SSU-G436, SSU-G867, SSU-U1804, LSU-G1303, LSU-A4541, and 5.8S-U14) exhibiting decreased rRNA methylation changes in both datasets.

### Comparison of Muscle and Myonuclear SNORD Expression and Corresponding Site-Specific Modification during MOV

**Figure 2.**
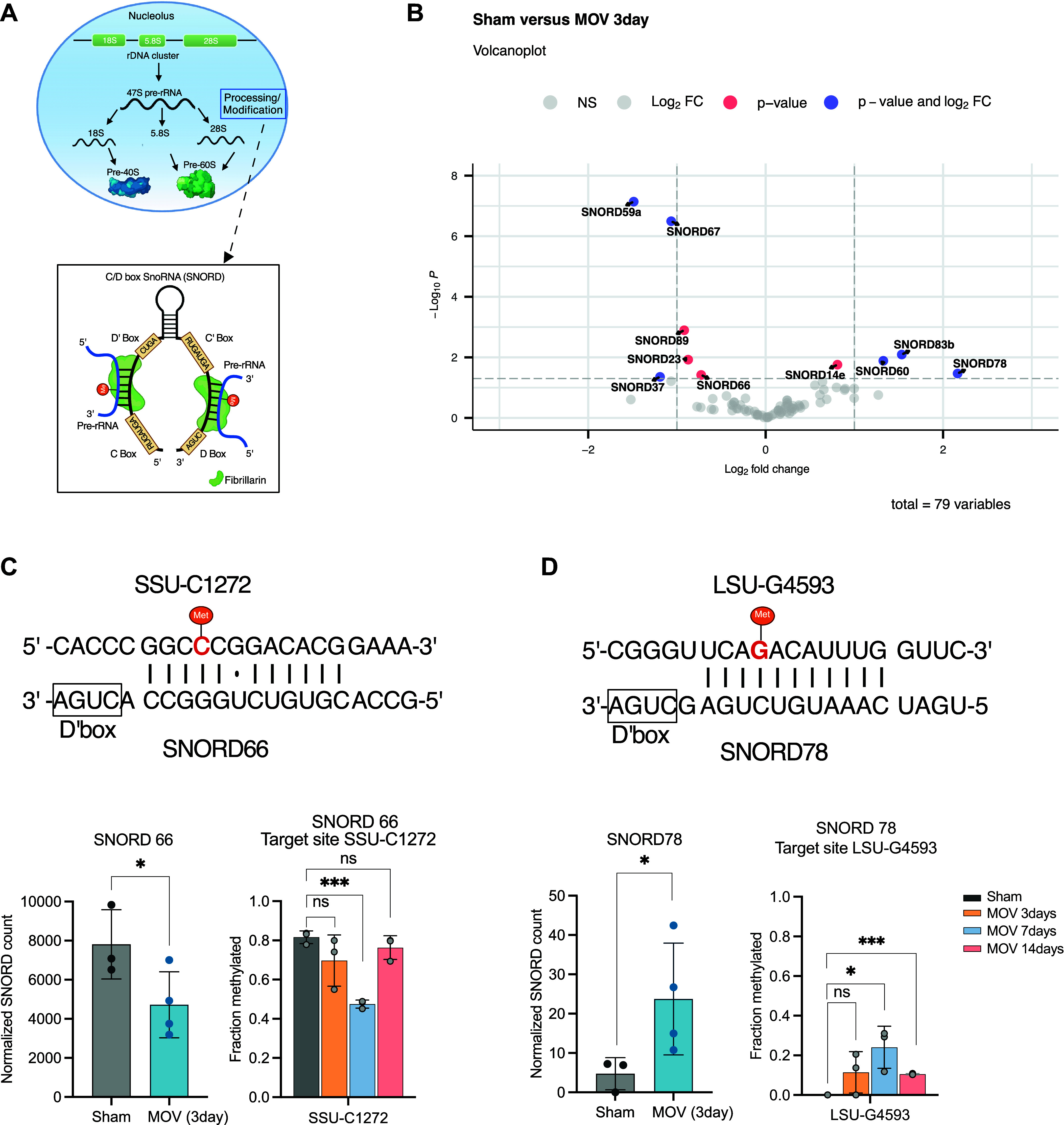
*A*: schematic representation of ribosomal RNA (rRNA) biogenesis and the formation of a box C/D small nucleolar RNA (SNORD)-rRNA-Fibrillarin complex in the nucleolus. *B*: volcano plot shows the fold change (*x*-axis) vs. the significance (*y*-axis) of the identified 79 SNORDs with adjusted *P* value <0.05 using Benjamini–Hochberg method and log2 fold change over 1. The significance (nonadjusted *P* value) and the fold-change are converted to −Log10 (*P* value) and Log2 (fold change), respectively. The vertical and horizontal dotted lines show the cutoff of fold change = ±1.0 and of *P* value = 0.05. The target sites corresponding to SNORDs and their host gene can be found in Supplemental Table S2. *C* and *D*: graph depicting selected SNORDs from volcano plot and matched to significant methylation sites from Fig. 1, *B–D* (*n* = 3 each time point) (*P* value <0.05). Bar graphs show normalized SNORD counts from RNA-Seq data of sham and 3-day overload [sham *n* = 3, mechanical overload (MOV) *n* = 4] (*P < 0.05, ***P < 0.001). Base pairing between the antisense element associated with box D′ SNORD and the target rRNA sequence that results in methylation of specific sites (labeled in Met). Created with BioRender.

The protein Fibrillarin (Fbl) directs site-specific 2′-*O*-Me on the transcribed rRNA as guided by SNORD (Fig. 2*A*) (13). We previously observed that myonuclear *Fbl* mRNA levels increase in myonuclei following mechanical loading in vivo (log2FC 1.085, adj.*P* = 0.027) (21), consistent with Fbl protein localization to myonuclei of cardiomyocytes and *Drosophila* muscle undergoing hypertrophy (35, 36). Therefore, we assessed SNORD expression at multiple time points following MOV experiment using a low-coverage data set derived from the RiboMeth-seq analysis. First, we matched the seven overlapping-modified rRNA sites with their corresponding guide SNORDs on *days 3*, *7*, and *14* (Supplemental Fig. S2*A*). Based on matching SNORDs, we observed a large variance for SNORD expression levels, likely attributed to the low read depth. Nevertheless, changes in SNORD expression were clearly evident and appear to start early during the MOV process (Supplemental Fig. S2*A*).

To further investigate potential regulatory steps upstream of the changes in site-specific rRNA modifications during the early phase of MOV, we analyzed myonuclear SNORD expression by small RNA sequencing following 3-day MOV, identifying 79 known SNORDs among in total 404 genes. Ten of these SNORDs were significantly different between sham and 3-day MOV (SNORD14e, 23, 37, 59a, 60, 66, 67, 78, 83b, and 89) (Fig. 2*B*). Their target sites and host genes are listed in Supplemental Table S2. Two sites with significantly altered rRNA 2′-*O*-Me were matched by significant changes in SNORD expression (Fig. 2, *C* and *D*), whereas the remaining SNORDs were not aligned with methylation changes at their target sites (Supplemental Fig. S2, *B*–*E*). One of the sites, SSU-C1272 was hypomethylated on *day 7* and its corresponding guide RNA, SNORD66, had a corresponding lower expression preceding this event (Fig. 2*C*). Moreover, LSU-G4593 was identified as the only site that exhibited a significantly higher 2′-*O*-Me levels, and this change was also found to be paralleled by elevated expression levels of its corresponding guide RNA SNORD78 (Fig. 2*D*). This is consistent with previous work demonstrating that this site is lowly methylated in fully differentiated cells but elevated upon proliferation (11, 37).

### Mechanical Overload Alters the Codon Composition Landscape of Protein-Coding Transcripts in Skeletal Muscle

Previous work by Jansson et al. (16) demonstrated that changes in rRNA 2′-*O*-Me at individual sites results in ribosomes that show translational preference for specific mRNA codons. Therefore, we assessed codon composition of protein-coding transcripts in skeletal muscle in response to 3-day MOV with the aim of exploring a potential temporal link between greater ribosome heterogeneity and an altered codon composition landscape. First, we reanalyzed protein-coding transcripts from previously published RNA-Seq data of 3-day MOV (21) and sham plantaris muscles to identify DEGs (Supplemental Fig. S3*A*). We previously demonstrated that extracellular matrix (ECM) gene expression is highly regulated during the acute phase of MOV (21, 38) and accompanied by a functional reduction in mitochondria respiration (18). Building upon our previous findings, additional pathway analysis indicated that ECM and respiratory electron transport (ETC) were among the top five pathways showing upregulation and downregulation (Supplemental Fig. S3*B*). Hence, we retrieved the coding sequence of differentially expressed transcripts belonging to each of these two gene ontologies, quantified the frequency of each codon among those transcripts, and compared it with the codon frequency of background genes (all protein-coding transcripts detected) (Supplemental Fig. S3*C*). This analysis revealed that certain codons are particularly more or less frequent in transcripts related to ECM organization, whereas other codons seem to be more or less frequent in transcripts related to ETC. Moreover, transcripts related to ECM organization seem to have higher GC-content (Supplemental Fig. S3*D*). Supplemental Figure S3*E* shows that the top five codons with significantly different frequencies in transcripts are associated with ECM and ETC. We noted that the codon *cac* (Histidine) was the only one following an opposite pattern in ECM- and ETC-related transcripts, being more frequent in ECM and less frequent in ETC (Supplemental Fig. S3*E*).

## DISCUSSION

During development, there is an increase in ribose methylation at many sites in rRNA across differentiated cell types (11, 17). The current study demonstrates that MOV of skeletal muscle reverts this pattern in skeletal muscle rRNA by triggering de novo synthesis of skeletal muscle ribosomes with a more “immature” methylation pattern that transiently diversifies the ribosome pool during the hypertrophic process. These findings are exciting as they identify epitranscriptic modifications of rRNA with potential implications for translational control in skeletal muscle at rest and during hypertrophic growth.

Accumulating evidence of fractionally methylated rRNA demonstrates the existence of distinct ribosome pools with differing 2′-*O*-Me patterns in varied cell types and emphasizes the importance of rRNA modifications to diversify the ribosome pool (4, 10, 11). Our data add to this body of work, as we observed several sites with fractional 2′-*O*-Me in both whole muscle rRNA and in immunoprecipitated ribosomes from the skeletal muscle-specific RiboTag mice. Thus, the presence of fractionally methylated sites strongly suggests ribosome heterogeneity in unperturbed skeletal muscle.

At the early stages of MOV, we observed a general reduction in 2′-*O*-Me of rRNA at multiple sites, resulting in a higher heterogeneity of the ribosomes in skeletal muscle (Fig. 1*E*). These findings were further substantiated by the presence of 2′-*O*-Me changes of rRNA in myofiber-specific ribosomes in response to MOV. Meanwhile, we used myonuclear-specific small RNA-Seq to determine SNORDs expression (Fig. 2*B*). We provide the first evidence showing that MOV alters the expression of myonuclear SNORDs, which is associated with changes in 2′-*O*-Me of skeletal muscle rRNA. This finding reveals the emergence of a nascent pool of ribosomes that contain a unique pattern of 2′-*O*-Me compared with the preexisting, homeostatic ribosomes. To the best of our knowledge, this is the first study presenting evidence of a clear change in the rRNA methylation pattern in response to an external stimulus in a fully differentiated tissue. Previous work has highlighted that during development, multiple tissues, including the brain, undergo a general increased methylation of the rRNA subunits, but that several sites remain fractionally methylated (11, 17, 39). Fractionally methylated positions have previously been suggested to be bona fide candidates for physiological regulation, possibly in a cell-type and/or developmental-stage manner (10, 40, 41). Thus, our study points toward the contribution of ribosome heterogeneity at the level of posttranscriptional nucleotide modifications during skeletal muscle hypertrophic growth.

Although the upstream pathways governing changes in the levels of individual rRNA 2′-*O*-Me sites are not fully understood, there is evidence showing that site-specific rRNA methylation responds to changes in the level of MYC expression, which in turn influences the translation of distinct mRNAs (16). Also, MYC induction in fibroblasts elevates the expression of SNORD45C, which acts as the guide RNA promoting SSU-C174 methylation (16). Thus, SNORD45C knockout cells completely lacked 2′-*O*-Me at SSU-C174. Depending on the methylation status of the SSU-C174 site, ribosomes were observed to translate certain mRNAs at different efficiencies. Specifically, based on codon composition, mRNA transcripts with a high frequency of GC-rich codons were translated more efficiently and mRNA transcripts with a high frequency of AU-rich codons less efficiently in the absence of methylation at the SSU-C174 site (16). Based on the hypothesis that increased ribosome heterogeneity could be a skeletal muscle response to an altered codon composition landscape during early stages of skeletal muscle hypertrophy, we analyzed previously published data on protein-coding transcripts following 3-day MOV (21). Interestingly, codon composition differed significantly between mRNAs of upregulated versus downregulated DEGs and differed with respect to GC-content (Supplemental Fig. S3, *C*–*E*). This analysis has limitations in drawing definitive conclusions, but it provides a basis for further investigation into the relationship between specialized ribosomes and different codon compositions during MOV. Further in-depth study will be required to understand how ribosomes with differing molecular phenotype, e.g., through 2′-*O*-Me changes, affect translation, regarding potential differences in translation based on codon compositions. The hypertrophic model used in this study puts a marked mechanical load on the muscle and is associated with inflammation and regeneration, suggesting the need for additional experimentation in less severe models to further establish the role of ribsome heterogeneity in skeletal muscle hypertrophy.

This is the first study to provide evidence of skeletal muscle ribosome heterogeneity at the myofiber level with respect to 2′-*O*-Me patterns both at rest and specifically as a response to mechanical overload. Furthermore, we present data that suggest newly made ribosomes during the early phase of hypertrophic growth are different as compared with typical myofiber ribosomes, which opens the possibility of special ribosome requirements during skeletal muscle hypertrophy.

## DATA AVAILABILITY

Raw data from Ribometh-seq experiments are available in Gene Expression Omnibus GEO273053.

## SUPPLEMENTAL MATERIAL

10.6084/m9.figshare.25943965 Supplemental Figs. S1–S3 and Supplemental Tables S1 and S2: https://doi.org/10.6084/m9.figshare.25943965.

## GRANTS

Funding to H.N. was from the Carlsberg Foundation and the NEYE-Foundation for establishment of a sequencing facility. Y.W. was supported by grants from the National Institute of Health K99 AR081367. K.A.M. was supported by the National Institutes of Health Grant R00 AG063994. I.J.V. was supported by the National Institutes of Health Grant P20GM104320-07. M.C. was supported by the Chinese Scholarship Council. The study was also supported by the Swedish research council under Grant No. 2022-01392, AFM-Telethon under Grant No. 23137, Åke Wiberg, Swedish Medical Association, and the Swedish Research Council for Sport Science Grant 2022/10, 2023/09 (to F.v.W.). T.S. and F.v.W. have also been supported by research fund to the Center for Neuromusculoskeletal Restorative Medicine from Health@InnoHK program launched by Innovation and Technology Commission, the Government of the Hong Kong Special Administrative Region of the People’s Republic of China.

## DISCLOSURES

Y.W. is the founder of Myoanalytics LLC. None of the other authors has any conflicts of interest, financial or otherwise, to disclose.

## AUTHOR CONTRIBUTIONS

H.N. and F.v.W. conceived and designed research; M.H., V.C.F., Y.W., N.K., H.N., and F.v.W. performed experiments; M.C., P.J., M.H., V.C.F., Y.W., I.J.V., N.K., B.J., H.N., and F.v.W. analyzed data; M.C., P.J., V.C.F., Y.W., I.J.V., B.J., S.E., J.L., J.M., K.A.M., T.S., H.N., and F.v.W. interpreted results of experiments; M.C., P.J., and S.E. prepared figures; M.C. drafted manuscript; M.C., P.J., M.H., V.C.F., Y.W., I.J.V., N.K., B.J., S.E., J.L., J.M., K.A.M., T.S., H.N., and F.v.W. edited and revised manuscript; M.C., P.J., M.H., V.C.F., Y.W., I.J.V., N.K., B.J., S.E., J.L., J.M., K.A.M., T.S., H.N., and F.v.W. approved final version of manuscript.
